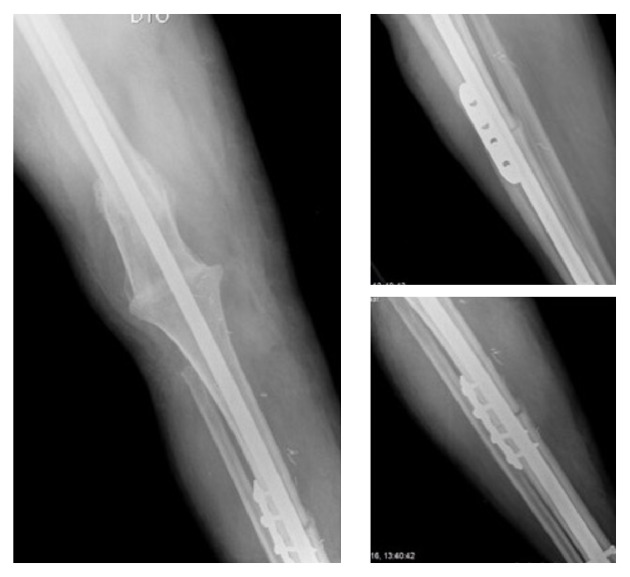# Lower Limb Reconstruction with Tibia Allograft after Resection of Giant Aneurysmal Bone Cyst

**DOI:** 10.1155/2016/7980593

**Published:** 2016-06-20

**Authors:** Joaquim Soares do Brito, Joana Teixeira, José Portela

**Affiliations:** Orthopaedics and Trauma Department, Centro Hospitalar Lisboa Norte, EPE-Hospital de Santa Maria, 1649-036 Lisboa, Portugal

## Abstract

Aneurysmal bone cysts (ABCs) are benign, expansible, nonneoplastic lesions of the bone, characterized by channels of blood and spaces separated by fibrous septa, which occur in young patients and, occasionally, with aggressive behavior. Giant ABC is an uncommon pathological lesion and can be challenging because of the destructive effect of the cyst on the bones and the pressure on the nearby structures, especially on weight-bearing bones. In this scenario,* en bloc* resection is the mainstay treatment and often demands complex reconstructions. This paper reports a difficult case of an unusual giant aneurysmal bone cyst, which required extensive resection and a knee fusion like reconstruction with tibia allograft.

## 1. Introduction

Aneurysmal bone cyst (ABC) is a rare benign bone tumor that contains blood-filled cavernous spaces separated by septa containing osteoid tissue and osteoclast giant cells [1–3]. Young patients are most often affected with tumors located in long bones metaphysis. In less frequent occasions we can find ABC in pelvis and spine, and sometimes ABC could be present with aggressive behavior [1–3].

Several treatment modalities have been described for ABC such as sole curettage, curettage with cementation or bone grafting, fibrosing agents or bone marrow injections, arterial embolization, adjuvant cryotherapy or radiotherapy, demineralized bone matrix applications, and segmental or* en bloc* resections [4]. Small lesions with minimal destruction or expansion of cortical bone can be treated with intralesional procedures with or without bone grafting; however, aggressive large-sized and expansible tumors should be treated through segmental or* en bloc* resection techniques and reconstruction with structural grafts [5].* En bloc* resection has the additional advantage of allowing obtaining the lowest association with recurrence which is as low as 0% [6–8]. However, resection can be problematic, especially for the lesions located in functionally important segments, when the tumor is unusually large or in the presence of concomitant pathological fracture.

It is not common to find the term “giant aneurysmal bone cyst” in the literature, mainly because it is considered a benign tumor. Nonetheless, some lesions can reach remarkable sizes, particularly if not treated properly allowing their growth through time [9]. Herein we present an unusual clinical case of a giant aneurysmal bone cyst located in proximal tibia, which eventually evolved to a supracondylar femur pathological fracture. Our surgical strategy was supported on a large resection and lower limb reconstruction with knee fusion using a tibia structural allograft. One year after surgical procedure, the patient is functionally independent, without walking aid and with minimal limb length discrepancy.

## 2. Clinical Case

**Figure 1:**
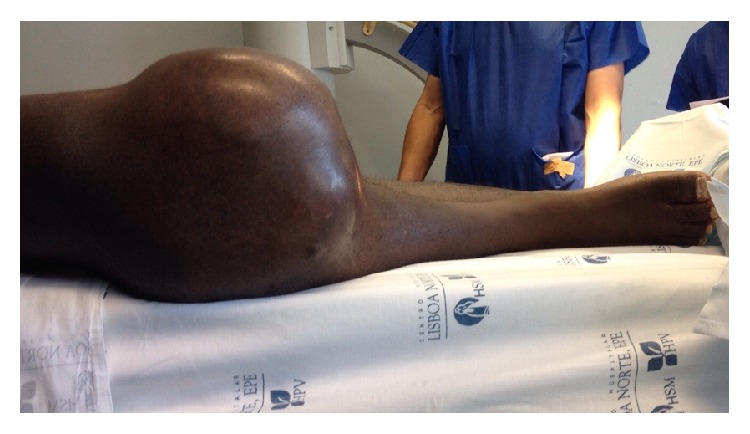


A 25-year-old black man originally from Guiney presented in the emergency department with a four-year history of a right knee slow growing mass for evaluation. The patient had a giant mass located around the right knee, which was in forced flexion and with no extension ability. There was no pain or vascular or neurological compromise despite the remarkable size of the lesion. Nonetheless, patient could not walk due to tumor size and knee fixed flexion (Figure 1). No other clinical findings or associated symptoms were disclosed.

**Figure 2:**
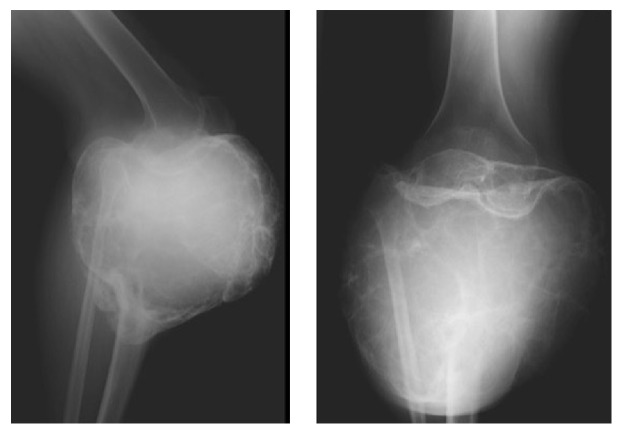


**Figure 3 fig3:**
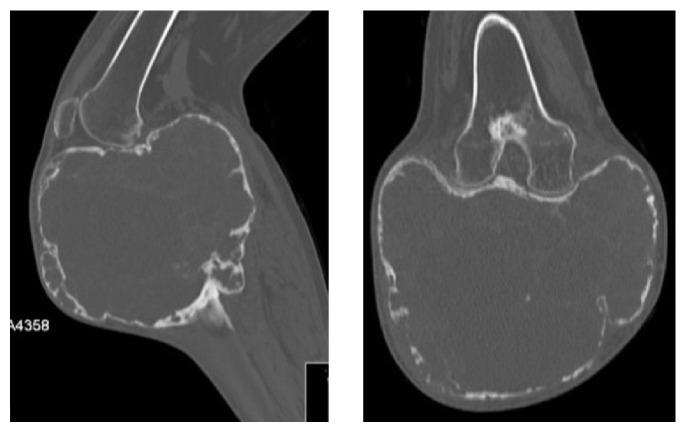


**Figure 4 fig4:**
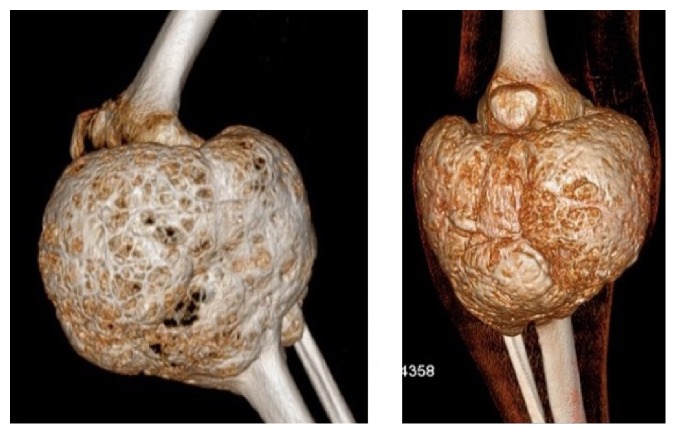


**Figure 5:**
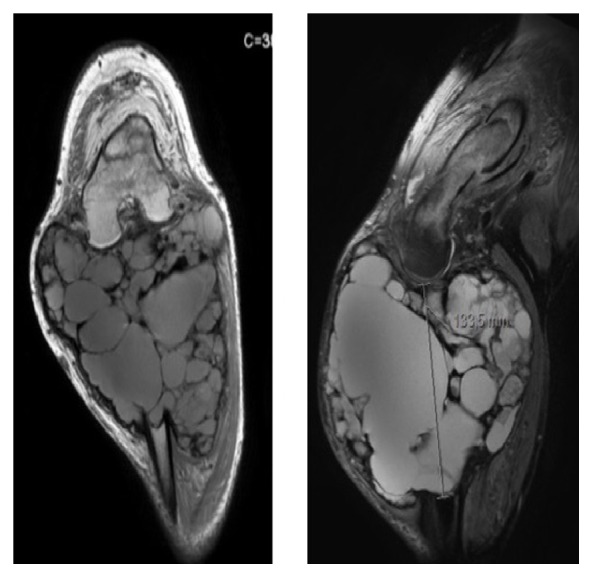


The X-ray, CT scan, and MRI (Figures 2 [Fig fig3] [Fig fig4]–5) revealed images showing an unusual large bone tumor of the proximal tibia. The patient underwent bone biopsy for definitive histological diagnosis, which was consistent with giant cell tumor. A radical surgical resection was proposed.

Preoperatively the patient returned to the emergency department due to a low energy fall but with an excruciating pain in the right knee. The new X-ray series disclosed a supracondylar femur fracture requiring surgery.

**Figure 6:**
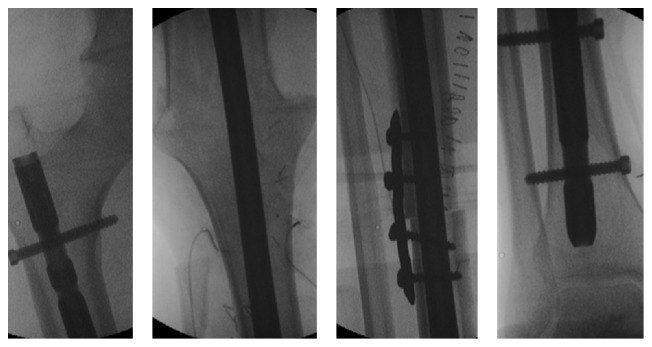


To obtain a most secure solution regarding a patient originally from Guiney where there is no medical assistance, we chose to perform an extensive extra-articular* en bloc* resection (until the supracondylar fracture site) and reconstruct the lower limb as a knee fusion, with a tibia structural allograft. Intraoperatively we performed an extra-articular resection through the supracondylar femur segment and the tibia diaphysis. All nerves and blood vessels were preserved. A tibia allograft was then interposed in the defect, and a knee arthrodesis nail was used to stabilize the construct (Figure 6). Distally, the tibia allograft received the additional support of plate and screws to increased integration probabilities within the remaining patient's tibia.

**Figure 7:**
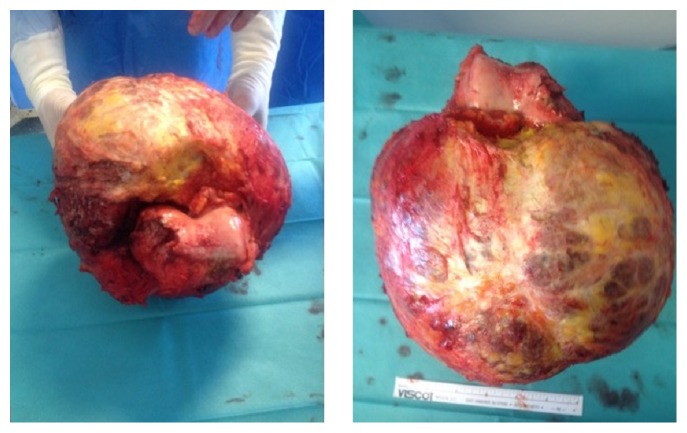


Postoperatively there were no complications, which allowed patient discharge during the first week. Partial weight bearing supported by crutches was allowed since the first day after operation. Follow-up with clinical and radiographic evaluation took place in the outpatient clinic, again without complications. The final histopathology diagnosis of the specimen (Figure 7) was an aneurysmal bone cyst.

Currently, with one year after the index operation, the patient is independent for daily live activities and only uses a walking aid occasionally. Radiographic assessment revealed no construct failure and good evolution to allograft integration (Figure 8). Limb length discrepancy is about two centimeters with no impact in function, which will allow patient to return to his home country.

## 3. Discussion

Aneurysmal bone cysts (ABCs) are often found in long bones metaphysis; nonetheless, they could be present in any other location, as the vertebral column and pelvis [1–5]. ABC can be present early before reaching giant size, which facilitates early diagnosis and treatment. It is well known that ABC is classified as an aggressive benign bone tumor, which means that if not treated properly, it may recur or if left untreated, it may get larger and eventually grow to be a giant ABC [9]. These aggressive lesions are difficult to address and could be challenging to any orthopaedic surgeon.

Although the pathogenesis of ABCs is still unknown, they could be considered either primary (70%) or secondary (30%) [5]. Primary ABCs arise* de novo*. A secondary ABC develops in association with other neoplasms most commonly giant bone tumor (GCT) of the bone, osteoblastoma, chondroblastoma, and fibrous dysplasia [10]. Radiographically, the diagnosis of an ABC shows five classic findings [3]. First, the neoplasm is typically present as an expansile lytic lesion with a soap-bubble appearance. Second, it presents an eccentric lesion outlined by a thin layer of subperiosteal new bone. Third, it presents a centric lesion. Fourth, it reveals a metaphyseal lesion that occupies a large percentage of the bone with trabeculations at the edges. Fifth, it manifests soft tissue expansion and destruction of the cortex. Additionally, it is suggested that if the cyst's transverse diameter on radiographic examination is equal to or more than three times the diameter of the adjacent normal bone, it can be called giant ABC [5]. Our patient fits these characteristics and by doing so we could considerer this particular lesion as a giant ABC.

Curettage and/or* en bloc* resection are treatments of choice for accessible lesions; meanwhile, other treatment modalities including percutaneous intralesional injection, cryotherapy, radiation, and embolization have been used for less accessible or recurrent lesions [5]. Chemical cauterization with phenol is recommended for large primary lesion to kill any surface tumor cells of the curetted cavity [3]. Cryotherapy has also been proposed as an adjuvant therapy with surgical treatment to achieve local control [11]. Radiation is used in inaccessible sites where no surgical options are available and selective arterial embolization is recommended as a procedure for lesions whose location or size makes other treatment modalities difficult or dangerous [3, 12–17]. Additionally, in large tumors similar to this case, arterial embolization could be a definitive treatment (even with serial embolizations) or used as adjuvant to the surgical technique, which allows improving surgical safety.

This particular case had three major hazards: the size of the lesion for one side, which demanded a wide resection; the articular involvement of the knee with loss of articular function; and finally a concomitant supracondylar femur fracture, which was an adverse event that represented an additional difficulty to limb reconstruction. In this setting, it is important to focus on the patient, who was originally from Guiney, a country where medical assistance is lacking. These facts and the patient returning home were important to the final treatment decision.

Large defects after resections of aggressive and giant aneurysmal bone cysts are difficult to treat. Various reconstructive options are available to fill these defects and provide bone integrity, including allogeneic or autogenic bone grafts and many different bony substitutes [5]. Our choice was to sacrifice the knee articulation, providing a knee fusion-like construct using a tibia allograft. This option was preferred for several reasons: firstly we were looking for a definitive solution with a life span, thinking in the probable revision surgery if we had a total knee replacement; secondly there were a lack of medical assistance in Guiney and an inability to return to periodic consultation; and finally we needed a permanent solution which allows high demand performance, according to a young rural worker.

Treatments for aneurysmal bone cysts should be individualized and take into account the location, aggressiveness, and extent of the lesion [5]. Cortical strut allografts have an important role in the treatment of large benign bone lesions after resection and bring the advantage of unlimited supply without additional donor site morbidity [18]. Meanwhile, the incorporating process of allografts is slower and probably less complete than that with autografts due to a low-grade immune response or a lack of osteocytes in the graft or both [19, 20]. Vascularized bone grafts have been suggested as the best method to replace large bone defects due to the ability for faster full incorporation and remodeling. Despite these advantages, vascularized bone grafts are technically demanding procedure and with a high failure rate for those without large experience [21–24]. Nonvascularized grafts are technically much easier to use and provide excellent structural bone support at the recipient side [21]. Successful long-term results of surgical* en bloc* resection and replacement with nonvascularized, autologous fibular, or tibial graft have already been reported in the literature [25]. Abuhassan and Shannak reported the results of nonvascularized fibular graft for the reconstruction of bone defects after* en bloc* resection of giant ABC in three patients [9]. They observed insufficient graft incorporation at the distal part of the fibular graft in the humerus case at the 18th month postoperatively. They treated this patient by open reduction and internal fixation with additional bone grafting and based on this experience advised rigid fixation of fibular graft onto the normal bone as a supplemental form of internal fixation to prevent graft insufficiency [9]. In the present case, the final construct obtained was stable and allowed progressive weight bearing without graft or osteosynthesis material failure. One year after surgery, the patient is independent and ready to return home.

## Figures and Tables

**Figure 8:**